# Aggregating Patient Safety and Status Information in the Electronic Health Record to Support Time-Sensitive Mobility Interventions in the Intensive Care Unit: Protocol for the Design and Testing of a Clinical Decision Support Tool

**DOI:** 10.2196/75752

**Published:** 2025-10-16

**Authors:** Anna Krupp, Heather Dunn, Lindsey Knake, Patrick Ten Eyck, Laura Frey-Law, Karen Dunn Lopez

**Affiliations:** 1 Acute and Critical Care Division College of Nursing University of Iowa Iowa City, IA United States; 2 Stead Family Department of Pediatrics, Division of Neonatology Carver College of Medicine University of Iowa Iowa City, IA United States; 3 Institute for Clinical and Translational Science University of Iowa Iowa City, IA United States; 4 Department of Physical Therapy and Rehabilitation Science Carver College of Medicine University of Iowa Iowa City, IA United States

**Keywords:** early mobility, early ambulation, critical care, nursing, nursing informatics, decision support systems, electronic health record

## Abstract

**Background:**

Patients who require intensive care unit (ICU) care frequently develop hospital-acquired functional decline, defined as a new or worsening loss of ability to perform self-care activities that is associated with prolonged immobility. This morbidity may persist for months to years after hospitalization but is potentially preventable through initiating mobility in the ICU using a multidisciplinary, evidence-based intervention to maintain functional status. While guidelines for ICU physical activity exist, timely identification of patients suitable for activity interventions is an ongoing challenge due to the dynamic nature of critical illness and the number of locations in the electronic health record (EHR) that clinicians need to click in and out of to synthesize patient data. Therefore, there is a critical need to develop an effective knowledge-based clinical decision support system (CDSS) interface in the EHR for efficient identification of patients appropriate for physical activity interventions and coordination of patient-specific activity plans within the ICU team.

**Objective:**

The objective of this 2-phase project is to develop a CDSS interface for consistent translation of patient-specific data to inform evidence-based physical activity interventions delivered by registered nurses and physical therapists in ICU settings and evaluate its usability, usefulness, cognitive workload, acceptability, feasibility, and effectiveness on decision-making outcomes.

**Methods:**

In phase 1, we will develop a usable, useful, and acceptable CDSS prototype by conducting 4 rounds of user-centered design interviews with registered nurses and physical therapists by using think-aloud and cognitive interview methods. In preparation for implementing CDSS in phase 2, we will conduct semistructured stakeholder interviews using the Consolidated Framework for Implementation Research to identify clinical workflow considerations, potential barriers, and implementation strategies. In phase 2, we will evaluate CDSS’s usability, cognitive workload, acceptability, and effectiveness for activity guideline adoption in two settings: (1) a simulated EHR environment and (2) two adult ICU units in a tertiary care hospital.

**Results:**

This study received funding in April 2024. The CDSS development phase is expected to conclude by December 2025. Data collection and analysis of CDSS evaluation are expected to begin in April 2026 and conclude by December 2028.

**Conclusions:**

We expect the results of this multimethod process for designing, testing efficacy, and identifying barriers to real-world use to have an important positive impact on others who seek to develop safe and effective CDSSs that align with clinical workflow. Importantly, this work will complete the necessary pilot study for a subsequent multisite pragmatic clinical trial to scale the concurrent use of patient data with guideline recommendations at the point of care to deliver evidence-based interventions to reduce hospital-acquired functional decline and its negative, costly outcomes.

**International Registered Report Identifier (IRRID):**

DERR1-10.2196/75752

## Introduction

### Background

New functional impairment after hospitalization for critical illness is common and persistent. More than 5 million adults are admitted to intensive care units (ICUs) each year in the United States, and more than 50% of them develop new physical health problems or hospital-acquired functional decline [1-4]. Functional decline is associated with negative outcomes during and after hospitalization, including ICU readmission, prolonged hospitalization, hospital readmission, nursing home admission, inability to return to work, and death [5-8]. These significant and long-term patient consequences demonstrate an urgent need to mitigate hospital-acquired functional decline.

**Figure 1:**
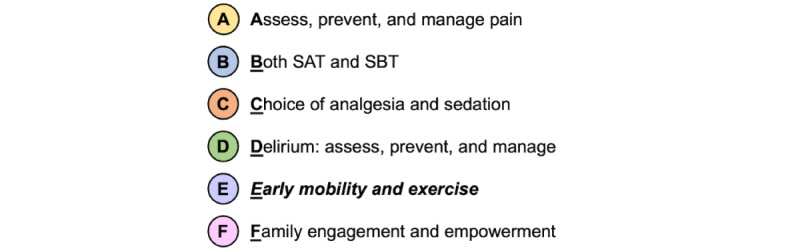
The ABCDEF bundle of standardized care. SAT: spontaneous awakening trial; SBT: spontaneous breathing trial.

The etiology of functional decline includes a combination of preexisting health status, the acute illness, and the consequences of ICU care (eg, deep sedation, immobility) [9,10]. The results of rigorous, well-designed studies demonstrate that early and routine physical activity interventions are safe and effective in improving functional recovery by the time of hospital discharge [11-13]. Multiple clinical practice guidelines recommend physical activity interventions as part of a bundle of standardized care for all patients in the ICU [14,15]. The ABCDEF bundle (Figure 1) was developed to standardize processes for coordinating and delivering evidence-based interventions in the ICU [16,17]. The early mobility (“E”) bundle element describes daily, progressive physical activity interventions for physiologically stable patients, beginning with exercises in bed, transferring to a chair, and advancing to walking. The results of ABCDEF bundle implementation studies demonstrate the benefits of increased frequency and level of physical activity, decreased ICU and hospital lengths of stay, and decreased mortality [18-20]. However, reliable implementation of all elements remains challenging. A national, 68-hospital quality improvement collaborative study routinely measured ABCDEF bundle implementation performance and found that mobility was the least frequently implemented bundle element, with only 29% of patients receiving physical activity interventions beyond active range of motion [20]. To improve overall ABCDEF bundle implementation and patient outcomes, specific focus on innovative strategies to overcome barriers to physical activity is needed.

Numerous barriers to physical activity interventions have been identified and broadly classified as patient-, clinician-, unit-, protocol-, or ICU context–related barriers [21]. Strategies to address these barriers have included the use of structured quality improvement models to identify and target local barriers [22], nursing-initiated physical activity protocols to standardize patient assessment and goal setting [23], and focused interdisciplinary communication and collaboration [24,25]. However, these resources and approaches have not spread widely. A limitation to physical activity protocols is the poor usability, given the temporal and cognitive demands associated with the workflows of registered nurses (RNs). Our previous research identified that obstacles to existing RN-initiated physical activity protocols are significant—requiring time to synthesize multiple patient data elements either across different locations in the electronic health record (EHR; eg, blood pressure in vital signs documentation and vasopressor dose in the medication administration record) or scrolling through data over several hours or shifts to determine patient stability or changes in condition (eg, determining whether current ventilator settings represent an increased, decreased, or unchanged amount of respiratory support) [26].

Clinical decision support system (CDSS) interventions are particularly relevant in ICU settings to support decision-making and reduce cognitive load stemming from the large amounts of data from disparate areas of the EHR that clinicians must filter and process to make time-critical decisions [27,28]. CDSSs can provide data visualizations, alerts, reminders, dashboards, and decision support to augment clinicians in complex decision-making and support coordinated care delivery [29,30]. CDSSs can play an important role in the timeliness of physical activity decision-making, which significantly impacts patient outcomes. There is significant evidence that bed rest duration is associated with loss of muscle mass and strength and subsequent functional decline, which begins within 24 hours of bed rest and rapidly worsens, with a loss of up to 5% to 10% of muscle strength within 7 days of bed rest [31,32]. Nevertheless, there remains a lack of multidisciplinary EHR-integrated CDSSs designed to enhance critical thinking, promote guideline adoption, and decrease the cognitive burden of this complex decision-making process. In addition, compared to medical CDSSs, few CDSSs have been designed and tested for ICU RNs and even fewer for physical therapists (PTs) [33]. Given that physical activity interventions are delivered by multiple ICU team members, a CDSS is needed to support decision-making and physical activity care coordination across disciplines within the ICU team. Considering the low implementation of physical activity interventions during hospitalization, especially in ICU settings, it is imperative to identify methods to increase the adoption of evidence-based physical activity interventions.

### Study Objectives

The overall objective of this 2-phase project is to develop and evaluate a CDSS interface for consistent translation of patient-specific data to inform evidence-based physical activity interventions delivered by RNs and PTs in ICU settings. Our team has successfully developed and validated a knowledge-based algorithm to synthesize patient data from multiple areas of the EHR with physical activity guideline recommendations (Aggregating Patient Safety and Status Information to Support Time-Sensitive Interventions in the Intensive Care Unit [ASSIST-ICU]) [34]. In phase 1, we aim to (1) develop the ASSIST-ICU CDSS interface using user-centered design (UCD) interviews with RNs and PTs and (2) develop a plan for implementing CDSS in 1 health system by interviewing key stakeholders. In phase 2, we aim to evaluate the effectiveness of the ASSIST-ICU CDSS for increasing the use of evidence-based physical activity interventions in the ICU delivered by RNs and PTs in (1) a simulated EHR environment and (2) a real-world pragmatic trial. The phase 2 secondary objectives are to evaluate the usability, usefulness, cognitive workload, acceptability, and implementation feasibility of the ASSIST-ICU CDSS.

### ASSIST-ICU Algorithm Development

ASSIST-ICU is a knowledge-based algorithm using discrete nursing, respiratory therapy, and physical therapy documentation to synthesize patient data with physical activity guideline recommendations. The ASSIST-ICU algorithm was developed using an iterative process involving (1) translating expert recommendations into operational definitions, (2) identifying discrete data fields in the EHR, and (3) developing logic statements for the algorithm [35]. We used an expert consensus statement on early mobility readiness and safety criteria to develop operational definitions in 4 domains: respiratory, cardiovascular, neurological, and other [36]. An example from the respiratory domain is shown in Table 1. Each domain is evaluated using 24 hours of data to score a patient’s readiness for mobility (yes or no). Overall readiness is scored as “yes” only if all domains are scored as ready for mobility; if any domain is scored as “no,” the overall readiness score is “no.”

Our team established content validation of the operational definitions [37], and retrospective testing of the decision support rules has been published [34]. The ASSIST-ICU CDSS will be delivered via an EHR tool. Initial feedback from focus groups will inform prototype development to include an icon that indicates readiness for mobility (“yes” or “no”) with the ability to drill down to the features contributing to the score, providing details on why the patient met or did not meet the readiness elements.

**Table 1:** Example of algorithm development process from the respiratory domain.

Variables	Description
Expert recommendation [36]	Clinical considerations for maintaining respiratory safety with out-of-bed exercises include that an artificial airway (eg, endotracheal tube, tracheostomy tube) is not in itself a contraindication, and SpO_2_^a^ should be ≥90%
Operational definition	If a patient is receiving mechanical ventilation, the patient’s most recent SpO_2_ reading and the 24-hour average SpO_2_ reading must be ≥90%
Data fields	Medical device group=endotracheal tube or tracheostomy SpO_2_ row
Logic statement	If medical device group=endotracheal tube OR tracheostomy tube, then most recent SpO_2_ reading ≥90% AND 24-hour average SpO_2_ reading ≥90%

^a^SpO_2_: peripheral capillary oxygen saturation.

### Conceptual Framework

**Figure 2:**
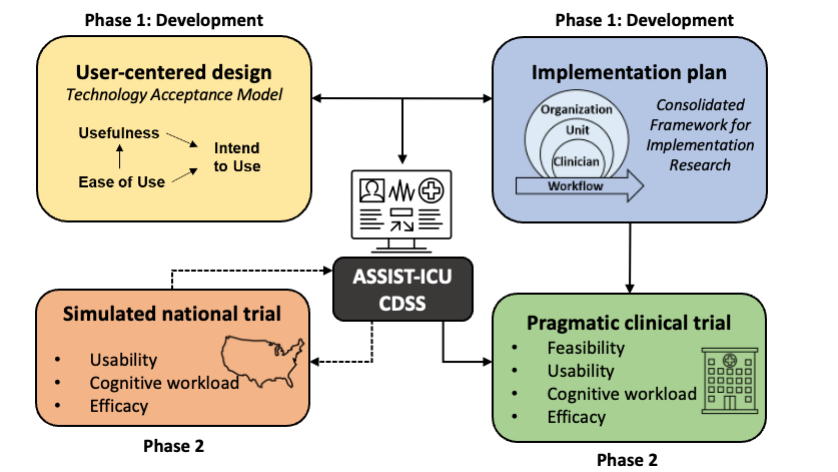
The 4 components of the study. ASSIST-ICU: Aggregating Patient Safety and Status Information to Support Time-Sensitive Interventions in the Intensive Care Unit; CDSS: clinical decision support system.

The theoretical basis for our study is grounded in an understanding of technology acceptance, cognitive load theory, and implementation science (Figure 2).

The technology acceptance model (TAM) is a widely used evidence-based model that focuses on perceived ease of use (or usability) and perceived usefulness. More than 30 years of research show that the TAM explains a substantial portion of health information technology acceptance and use [38,39]. Although multiple modifications of the TAM exist, we use the original TAM model for its parsimony, ease of use, and usefulness in explaining acceptance [40]. Cognitive load theory, a theory in use for more than 30 years, focuses on complex problem-solving when excessive cognitive load decreases decision-making performance [41,42]. A suboptimal CDSS design would increase extraneous cognitive workload and decrease a user’s ability to correctly interpret the evidence presented. Given the limited amount of information humans can process in working memory before experiencing cognitive overload, our CDSS will aim to minimize extraneous cognitive load and will be evaluated using the NASA Task Load Index (NASA-TLX) [43]. Technology use is also influenced by organizational factors that are addressed by implementation science. The Consolidated Framework for Implementation Research (CFIR) organizes 39 constructs across 5 domains: innovation, outer setting, inner setting, individual characteristics, and implementation process [44,45]. This study will focus primarily on the CFIR domains of inner setting (organization and unit), characteristics of individuals (clinicians), and implementation process.

## Methods

**Figure 3:**
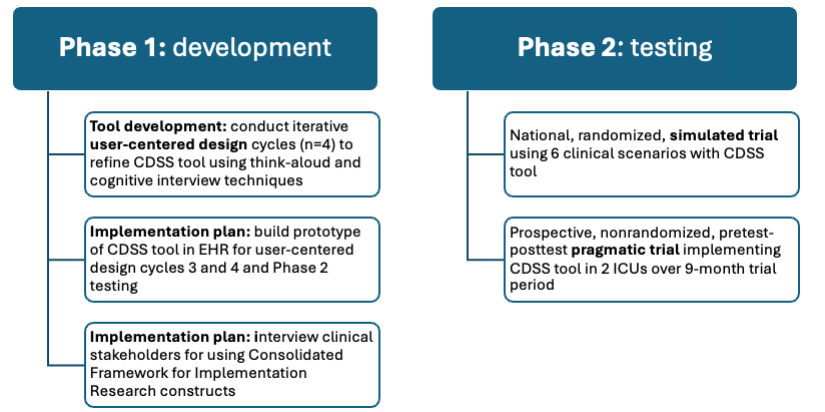
Details of the study’s 2 phases. CDSS: clinical decision support system; EHR: electronic health record; ICU: intensive care unit.

We will use a 2-phase approach using UCD and implementation science methodologies to develop a CDSS tool, implement the tool in 1 health system, and then test the tool in 2 settings (Figure 3).

### Phase 1: ASSIST-ICU CDSS Prototype Development and Implementation Plan

#### Iterative UCD Cycles to Design the ASSIST-ICU Prototype

We will design and iteratively revise the ASSIST-ICU CDSS prototype in a series of 4 UCD cycles with practicing critical care clinicians per cycle (n=24 RNs and PTs). We will conduct 2 types of sequential interviews—think-aloud [46] and cognitive [47] interviews—as well as a survey to assess acceptability (Multimedia Appendix 1) [38]. In the think-aloud interviews, participants will receive information about 2 patients with differing physical activity needs and will be instructed to open the prototype and navigate through the interface as they would if they were gathering clinical information in the EHR for making a clinical physical activity decision. As they explore the interface, they are instructed to vocalize about the actions they are taking and the thought processes they are using to interact with the prototype. This is a well-accepted usability method in which the clinician is not interrupted during their exploration. This method elicits users’ expectations, understanding, and misunderstanding and identifies usability problems [48]. To capture interpretations and features that may have been missed during the think-aloud interview, a cognitive interview will be conducted to assess clinician understanding and interpretation of the prototype features. In this method, we systematically review the prototype interface with the clinician, feature by feature, to assess whether the feature was interpreted as the designers intended as well as to solicit design ideas. Next, to assess acceptability, the participant will complete the TAM survey (Cronbach α=0.79-0.93) [49].

The design team will analyze the findings of the interviews by rating each feature as being positive, negative, or unclear using the following deductive coding categories: (1) ease of use, (2) usefulness, (3) interpretation, and (4) satisfaction. Features with ≥25% negative or unclear ratings will be redesigned [50]. Any redesign efforts will incorporate EHR feasibility into the methods to avoid unattainable solutions. After redesign, clinician interviews will resume with a new group of participants.

#### ASSIST-ICU CDSS EHR Integration

After completing the UCD process, we will collaborate with the clinical informatics team at our study site to build the final prototype of the ASSIST-ICU CDSS interface in their EHR training environment. To support simulated testing, we will create real-world simulated patients with simulated data to evaluate how the data will be displayed on the screen and how clinicians can interact with the data. Our CDSS design will aim to include primarily structured data fields that are already available in the EHR to minimize creating custom documentation fields to keep the design within a Fast Healthcare Interoperability Resources infrastructure that will allow for future interoperability between sites.

#### ASSIST-ICU CDSS Implementation Plan

We will gather input from key stakeholders to identify clinical workflow considerations, potential barriers, and implementation strategies for the ASSIST-ICU CDSS using a descriptive qualitative design, incorporating the CFIR to inform data collection and directed content analysis [44,45]. We will interview stakeholders using a semistructured interview guide (Multimedia Appendix 2). Interview questions will focus on (1) workflow considerations, (2) role needs, and (3) implementation strategies to prepare our team and participating site for the successful evaluation of CDSS. Questions will be tailored to key stakeholders (eg, ICU director of nursing, PT director, RN informaticist, and chief medical information officer), recognizing that individuals hold different levels of influence within the organization. Interview data will be analyzed using directed content analysis [26,51]. We will review findings from the interviews to identify a follow-up plan for overcoming potential barriers and addressing workflow considerations before the ASSIST-ICU CDSS clinical trial.

### Phase 2: ASSIST-ICU Simulated Trial Design

#### Overview

We will conduct a simulated trial comparing the ASSIST-ICU CDSS interface with usual care (no ASSIST-ICU interface) in a simulated EHR environment. We will operationalize evidence-based decision-making through the creation of 6 expert-validated clinical scenarios. Of the 6 simulations, 3 will meet the out-of-bed safety definition, and 3 will not. All 6 simulations will be completed by each participant in a random order generated using the RAND function of Microsoft Excel. The decisions (regarding whether out-of-bed activity is safe or unsafe) will be recorded, and the number of correct decisions (range 0-6) for each participant will serve as our outcome measure of effectiveness (Table 2).

**Table 2:** Operationalization for scoring each clinical scenario.

	ASSIST-ICU^a^ CDSS^b^ recommends patient out of bed: yes	ASSIST-ICU CDSS recommends patient out of bed: no
Clinician decision to mobilize patient out of bed: yes	1	0
Clinician decision to mobilize patient out of bed: no	0	1

^a^ASSIST-ICU: Aggregating Patient Safety and Status Information to Support Time-Sensitive Interventions in the Intensive Care Unit.

^b^CDSS: clinical decision support system.

#### Study Participants for Simulated Trial

The inclusion criteria will be as follows: (1) licensed RN or PT, (2) at least 6 months of experience working in an ICU (to align with the advanced beginner stage of the Dreyfus model of skill acquisition [52,53]), (3) access to a tablet or computer for the design session, and (4) fluent in English. The exclusion criteria will be as follows: (1) no EHR experience in a critical care setting and (2) inability to use a cloud-based video platform.

We will recruit a sample of 50 licensed RNs and PTs (control group: n=25, 50%; intervention group: n=25, 50%) using publicly available lists from the professional state boards of 7 geographically diverse states. We will randomly select clinicians to contact. Using a modified version of the tailored design method proposed by Dillman et al [54], each selected clinician will be contacted using different modes (ie, letters, postcards, and email) up to 5 times in a 21-day cycle. For generalizability purposes, we will use a quota sampling methodology to ensure that the clinicians represent the demographics of the profession [50]. In addition to the random sample, up to 15 clinicians will be recruited using convenience sampling to assess the fidelity of ASSIST-ICU (eg, agreement between clinical scenario sequence and variable display in CDSS) in the simulated EHR.

#### Sample Power for Simulated Trial

The primary hypothesis to be tested in this study focuses on the efficacy of ASSIST-ICU in making evidence-based guideline physical activity decisions in a simulated EHR environment compared to no decision support. We hypothesize that participants assigned to the ASSIST-ICU intervention group will achieve a higher proportion of decisions that meet the out-of-bed safety definition. Assuming a true mean of 4.5 correct decisions out of 6 simulations (75% probability) when using the decision tool and 3 correct decisions (50% probability) when not using it, 2-sample binomial test simulations show that 25 clinicians per group will provide 99.2% power when testing for a between-group difference at α=.05.

#### Study Procedures and Setting

Respondents who meet the eligibility criteria will participate in an informed consent process and complete a short demographic and clinical experience survey. At the time of the scheduled test session, participants will connect via the cloud-based video platform from their homes or a quiet space of their choosing. Each participant will connect with a member of the research team and share their screen as they interact with the prototype. This will allow the visualization of computer mouse movements to show which areas and features of the prototype they are focusing on. After receiving a standardized orientation to the simulated EHR environment, the research team member will begin recording, and the clinicians will open the simulated EHR environment (with or without the ASSIST-ICU CDSS) to begin the study. The fictional patients’ scenarios will be presented in their preassigned random order. After exploring the EHR environment, participants will be asked to respond whether they (1) would or (2) would not decide to move each patient out of bed that day.

#### Data Collection and Analysis for Simulated Trial

Clinicians will receive a score ranging from 0 to 6 based on the number of scenarios answered correctly. Participants will complete 3 brief surveys using a hyperlink in the cloud-based video platform’s chat feature to assess the usability (System Usability Scale [SUS] [55]), cognitive workload (NASA-TLX [43]), and acceptability (Acceptability of Intervention Measure [AIM] [56]) of CDSS and decision-making process as a whole. The SUS is a 10-item Likert-type scale that is widely used to measure usability across a broad range of interfaces and performs well psychometrically (Cronbach α=0.70-0.92) [57,58]. The NASA-TLX is a 6-item scale that measures subjective cognitive workload in performing a specific task and is a reliable measure of cognitive workload across industries, including ICU clinicians (Cronbach α=0.83-0.92) [59]. The AIM is a 4-item Likert-type scale that measures acceptability and performs well psychometrically (Cronbach α=0.85-0.91).

Summary statistics will be calculated for the collected measures by group. Continuous measure distributions will be assessed for normality and reported as means and SDs or medians and IQRs, as appropriate. Categorical measures will be reported as counts and percentages for each level. A 2-sample binomial test will be used to test the null hypothesis that decision accuracy with the support tool does not differ from accuracy without it. Further between-group assessments will be conducted for SUS, NASA-TLX, and AIM scores using 2-sample 2-tailed *t* tests or the Wilcoxon rank sum test. *P* values will be reported based on 2-sided alternatives, and statistical significance will be evaluated at α=.05.

### Phase 2: ASSIST-ICU Pragmatic Trial Design

The ASSIST-ICU pragmatic trial will use a prospective, nonrandomized, 2-unit pretest-posttest design. The specific components of the ASSIST-ICU CDSS and how the implementation will be operationalized, implemented, measured, and monitored for feasibility will be incorporated into the research protocol at the end of phase 1.

#### Study Setting and Participants for Pragmatic Trial

This study will take place at University of Iowa Health Care. Within University of Iowa Health Care, we will include 2 adult ICUs. Both units have implemented comprehensive patient physical activity programs that include protocol-based activity progression, routine PT consultation on ICU admission, and standardized activity assessment and intervention documentation for RNs and PTs in the EHR. The ASSIST-ICU CDSS will include 2 participant groups: clinicians and patients. The clinicians will include all RNs and PTs working in the selected adult ICU units. RN-to-patient ratios in the ICUs are typically 1:2, and PTs usually manage 6 to 8 patients per day. Patients will include all adults admitted to a participating ICU for at least 24 hours. As a pragmatic trial focused on decision-making at the point of care, we will not place restrictions on age, comorbidities, severity of illness, or expected discharge outcomes.

#### Intervention

As the trial involves using a prospective, nonrandomized, 2-unit pretest-posttest design, the ASSIST-ICU CDSS will be implemented in each ICU for a period of 9 months using a staggered approach, with 1 ICU receiving the intervention at a time. A training period will be conducted with RN and PT end users before implementation to facilitate effective use of the ASSIST-ICU CDSS and integration into existing workflows. Training will be scheduled based on each clinician’s schedule and last approximately 15 minutes. In addition, resource materials will be distributed to each unit before and throughout the trial.

#### Data Collection and Analysis for Pragmatic Trial

Feasibility and effectiveness data will be captured via the EHR. Study data will be extracted from the EHR using EHR reports that are built from the structured data fields used to create the ASSIST-ICU CDSS. Baseline data will be collected via the reports before the intervention implementation using silent data collection. Silent data collection functionality allows CDSS logic to actively run in the system, but users do not yet have access to use the CDSS dashboard, allowing for baseline data collection.

We will evaluate key implementation and clinical outcomes by using the RE-AIM (reach, effectiveness, adoption, implementation, and maintenance) framework because it considers both the feasibility (reach, adoption, and implementation) and clinical effectiveness outcomes of the intervention (Table 3) [60].

The primary feasibility outcome will be physical activity decision-making (defined as the number of patients with out-of-bed activity documented and a corresponding CDSS designation of “safe” on a given ICU day). Secondary measures include acceptability and usability of CDSS. Acceptability (defined as the belief that an intervention is agreeable, palatable, or satisfactory [56]) and usability (defined as being appropriate for a specific context of use [55]) will be measured through online surveys. We will also evaluate potential safety events routinely reported in physical activity studies [61] and documented in the health system’s patient safety reporting system, including whether the following events occurred during out-of-bed activity: fall to the floor, unplanned extubation, or line removal requiring urgent replacement.

On trial completion, all ICU RNs and PTs who worked in a study ICU will be invited to participate in a web-based survey. Usability (SUS), cognitive workload (NASA-TLX), and acceptability (AIM) will be measured and calculated in the same manner as described for the simulated trial.

We will generate histograms and bar charts to evaluate the distributions of all variables of interest and report appropriate descriptive statistics. Continuous variables will be summarized by means and SDs or medians and IQRs, as appropriate. Categorical variables will be summarized as counts and percentages. Preexisting differences in the outcome variables at baseline will be assessed using 2-sample *t* tests, the Wilcoxon rank sum test, or the Fisher exact test. Frequencies and percentages will be calculated to determine the feasibility of participation measures. A 2-sample binomial test will be used to test the null hypothesis that decision accuracy with the support tool does not differ from accuracy without it. Unit-stratified means and SDs will be calculated for SUS, NASA-TLX, and AIM scores. *P* values will be reported, and statistical significance will be evaluated at α=.05.

**Table 3:** Study outcomes by RE-AIM (reach, effectiveness, adoption, implementation, and maintenance) element.

Elements and end points		Data collection
**Reach**		
	80% of unit RNs^a^ and PTs^b^ participate in training	Unit participation logs
	90% of unit RNs and PTs participate in trial	EHR^c^ report
	80% of unit RNs and PTs complete postimplementation surveys	REDCap^d^
	100% of eligible patients receive CDSS^e^ recommendations in participating ICUs^f^	EHR report
**Effectiveness**		
	Ratio of safe alerts to total alerts	EHR report
	Primary clinical end point: time to first out-of-bed activity	EHR report
	Secondary clinical end points: highest level of activity in ICU, ICU and hospital lengths of stay, and discharge disposition	EHR report
**Adoption**		
	Number of tool views	EHR audit log
**Implementation**		
	Primary feasibility outcome: physical activity decision-making	EHR report
	Safety: fall to the floor and accidental tube dislodgement (endotracheal or line removal)	Safety reporting system
**Maintenance**		
	Continued physical activity practice after the clinical trial	EHR report

^a^RN: registered nurse.

^b^PT: physical therapist.

^c^EHR: electronic health record.

^d^REDCap: Research Electronic Data Capture.

^e^CDSS: clinical decision support system.

^f^ICU: intensive care unit.

### Ethical Considerations

Ethics approval has been obtained for phase 1 from the institutional review board (IRB) of the University of Iowa (202503553; May 15, 2025). The study received exempt status because it involves minimal risk and met the criteria outlined in the Code of Federal Regulations (45 CFR 46). All participants will receive a study information sheet and may opt out of the study at any time. Transcript data will be deidentified, and recordings will be stored in a password-secured research data storage service. Participants in the UCD interviews will receive a US $95 gift card as an honorarium.

For phase 2, we plan to seek IRB approval before beginning the clinical trial. We plan to submit an exempt application for the virtual trial, and participants will receive a US $95 gift card as an honorarium. For the ICU trial, we plan to request an IRB waiver of informed consent for patients admitted to the ICU and clinicians, based on the premise that the trial meets the Office for Human Research Protections regulations on waivers of consent (45 CFR 46.116[f](3)). Specifically, the research involves no more than minimal risk to the participants (patients and providers engaged in data collection procedures that pose minimal risk), and the CDSS intervention is designed to increase the uptake of evidence-based care.

## Results

This 2-phase CDSS development and evaluation study was funded in 2024 by the Agency for Healthcare Research and Quality (1R21HS029959-01). The developmental phase (phase 1) began in April 2024, and the CDSS trial is planned to begin in 2026, with data collection and analysis to be completed in April 2028. Phase 1 was approved by the IRB of the University of Iowa (202312543) with a waiver of informed consent, in accordance with the ethical standards of the institutional committee on human experimentation and the Declaration of Helsinki of 1975 and its latest updates. A separate IRB protocol will be submitted before beginning phase 2.

## Discussion

### Study Significance and Strengths

This study aims to develop and evaluate a CDSS that assists clinicians in coordinating and delivering evidence-based physical activity interventions at the point of care, with minimal extraneous cognitive workload and demonstrated feasibility of real-world trial procedures.

This study rigorously addresses the most common potential pitfalls identified with CDSSs, including usability, increased cognitive workload, usefulness, effectiveness, safety, fragmented workflows, and lack of spread, by using a novel sequence of methods to design and test the CDSS interface [29]. First, we use a UCD approach, considered a gold standard, which involves iterative evaluations with future system users to enable user perspectives to be incorporated into the system design to improve usefulness and usability [46,62-64]. Second, we use the CFIR implementation science framework during the design phase to understand barriers and facilitators to CDSS implementation [44,45]. Next, we conduct a virtual trial to establish the safety and effectiveness of the intervention in a simulated EHR environment before testing the feasibility of CDSS in real-world settings. Although UCD is widely used in health care, randomized tests of efficacy and rigorous testing of CDSSs in a pragmatic trial within a single study are rare. Furthermore, few CDSS studies apply implementation science theories to inform CDSS development and testing; however, designing with an implementation science lens can help identify additional modifiable barriers to address during CDSS design and implementation [65].

Despite the availability of multiple guidelines [14,36] and paper-based algorithms [23,66], there remains a lack of multidisciplinary EHR-integrated CDSSs designed to enhance critical thinking, promote guideline adoption, and decrease the cognitive burden of the complex mobility decision-making process regarding patients in the ICU. In addition, compared to medical CDSSs, few CDSSs have been designed and tested for ICU RNs and even fewer for PTs [33]. Given that physical activity interventions are delivered by multiple ICU team members, CDSSs are needed to support multidisciplinary decision-making and physical activity care coordination across disciplines within the ICU team. Considering the low implementation of physical activity interventions during hospitalization, especially in ICU settings, it is imperative to identify methods to increase the adoption of evidence-based physical activity interventions. ASSIST-ICU is expected to assist ICU clinicians in coordinating and delivering evidence-based physical activity interventions at the point of care, decrease bed rest duration, and ultimately reduce hospital-acquired functional decline.

### Study Limitations

Although our study team has expertise recruiting participants for a national, internet-based clinical trial of nurses, we recognize that recruiting a multiprofessional sample may be challenging. In our prior work, we successfully recruited a national sample using state nursing board lists and a modified version of the tailored design method proposed by Dillman et al [54] that includes repeated invitations with different methods (eg, postcard or mailed letter with a small cash token) [67]. In addition, we have assembled a team with representation from both nursing and physical therapy disciplines to help optimize our reach to both professions.

Barriers beyond the focus of CDSS (efficient patient identification and physical activity coordination) may impact patient physical activity practice. We envision that this intervention may help to identify and prioritize addressing other barriers because CDSS displays patient status at the unit level, which can support unit leadership decision-making (eg, staffing or equipment needs). Without identifying patient safety for physical activity, which is a fundamental step in planning physical activity interventions, secondary barriers, such as staffing, are more difficult to plan for and address. We have selected ICUs with existing ICU activity protocols and will track unit-level barriers that may impact physical activity practice; for example, if nursing units are experiencing high levels of turnover and temporary staffing, we are prepared to modify CDSS training as needed to support unit needs.

### Future Directions

The findings from this study will guide a future multisite pragmatic clinical trial to assist ICU clinicians in coordinating and delivering evidence-based physical activity interventions at the point of care. The successful completion of this study will be significant because CDSS is expected to ultimately be operable across different health systems (specified with Fast Healthcare Interoperability Resources standards), allowing for CDSS to be scaled across organizations and evaluated for clinical impact in a multicenter study. Future work will compare institutions’ current standard of care with the ASSIST-ICU visualization on patient outcomes and explore institution-specific implementation needs.

### Conclusions

Our study aims to develop a CDSS interface for consistent patient-specific translation of evidence-based physical activity interventions in ICU settings and evaluate its usability, usefulness, cognitive workload, acceptability, feasibility, and effectiveness on decision-making outcomes. We hypothesize that this novel interdisciplinary CDSS will increase the delivery of evidence-based recommendations for ICU physical activity. By applying UCD cycles and implementation science methods during development, we will seek to ensure that CDSS meets users’ needs at the point of care and is feasible for health systems to implement in existing clinical workflows. These methods may provide a framework for developing future interdisciplinary CDSSs. The insights gained from this study are expected to provide strong justification for a subsequent multicenter study to deliver evidence-based interventions aimed at reducing hospital-acquired functional decline and its negative, costly outcomes.

## Appendix

Multimedia Appendix 1 User-centered design interview guide.

Multimedia Appendix 2 Implementation planning interview guide.

## Abbreviations

AIM: Acceptability of Intervention Measure
ASSIST-ICU: Aggregating Patient Safety and Status Information to Support Time-Sensitive Interventions in the Intensive Care Unit
CDSS: clinical decision support system
CFIR: Consolidated Framework for Implementation Research
EHR: electronic health record
ICU: intensive care unit
IRB: institutional review board
NASA-TLX: NASA Task Load Index
PT: physical therapist
RE-AIM: reach, effectiveness, adoption, implementation, and maintenance
RN: registered nurse
SUS: System Usability Scale
TAM: technology acceptance model
UCD: user-centered design

## Data Availability

The datasets generated or analyzed during this study are available from the corresponding author on reasonable request.

## References

[1] Ferrante LE, Pisani MA, Murphy TE, Gahbauer EA, Leo-Summers LS, Gill TM (2016). Factors associated with functional recovery among older intensive care unit survivors. Am J Respir Crit Care Med.

[2] Ingraham NE, Vakayil V, Pendleton KM, Robbins AJ, Freese RL, Northrop EF, Brunsvold ME, Charles A, Chipman JG, Tignanelli CJ (2020). National trends and variation of functional status deterioration in the medically critically ill. Crit Care Med.

[3] Iwashyna TJ, Ely EW, Smith DM, Langa KM (2010). Long-term cognitive impairment and functional disability among survivors of severe sepsis. JAMA.

[4] Barrett ML, Smith MW, Elixhauser A, Honigman LS, Pines JM (2014). Statistical brief #185: utilization of intensive care services, 2011. Healthcare Cost and Utilization Project (HCUP) Statistical Briefs #185.

[5] Silveira LT, Silva JM, Tanaka C, Fu C (2019). Decline in functional status after intensive care unit discharge is associated with ICU readmission: a prospective cohort study. Physiotherapy.

[6] Covinsky KE, Pierluissi E, Johnston CB (2011). Hospitalization-associated disability: "she was probably able to ambulate, but I’m not sure". JAMA.

[7] Geense WW, Zegers M, Peters MA, Ewalds E, Simons KS, Vermeulen H, van der Hoeven JG, van den Boogaard M (2021). New physical, mental, and cognitive problems 1 year after ICU admission: a prospective multicenter study. Am J Respir Crit Care Med.

[8] McPeake J, Mikkelsen ME, Quasim T, Hibbert E, Cannon P, Shaw M, Ankori J, Iwashyna TJ, Haines KJ (2019). Return to employment after critical illness and its association with psychosocial outcomes. A systematic review and meta-analysis. Ann Am Thorac Soc.

[9] Hoenig HM, Rubenstein LZ (1991). Hospital-associated deconditioning and dysfunction. J Am Geriatr Soc.

[10] Needham DM, Wozniak AW, Hough CL, Morris PE, Dinglas VD, Jackson JC, Mendez-Tellez PA, Shanholtz C, Ely EW, Colantuoni E, Hopkins RO, National Institutes of Health NHLBI ARDS Network (2014). Risk factors for physical impairment after acute lung injury in a national, multicenter study. Am J Respir Crit Care Med.

[11] Schweickert WD, Pohlman MC, Pohlman AS, Nigos C, Pawlik AJ, Esbrook CL, Spears L, Miller M, Franczyk M, Deprizio D, Schmidt GA, Bowman A, Barr R, McCallister KE, Hall JB, Kress JP (2009). Early physical and occupational therapy in mechanically ventilated, critically ill patients: a randomised controlled trial. Lancet.

[12] Morris PE, Berry MJ, Files DC, Thompson JC, Hauser J, Flores L, Dhar S, Chmelo E, Lovato J, Case LD, Bakhru RN, Sarwal A, Parry SM, Campbell P, Mote A, Winkelman C, Hite RD, Nicklas B, Chatterjee A, Young MP (2016). Standardized rehabilitation and hospital length of stay among patients with acute respiratory failure: a randomized clinical trial. JAMA.

[13] Morris PE, Goad A, Thompson C, Taylor K, Harry B, Passmore L, Ross A, Anderson L, Baker S, Sanchez M, Penley L, Howard A, Dixon L, Leach S, Small R, Hite RD, Haponik E (2008). Early intensive care unit mobility therapy in the treatment of acute respiratory failure. Crit Care Med.

[14] Devlin JW, Skrobik Y, Gélinas C, Needham DM, Slooter AJ, Pandharipande PP, Watson PL, Weinhouse GL, Nunnally ME, Rochwerg B, Balas MC, van den Boogaard M, Bosma KJ, Brummel NE, Chanques G, Denehy L, Drouot X, Fraser GL, Harris JE, Joffe AM, Kho ME, Kress JP, Lanphere JA, McKinley S, Neufeld KJ, Pisani MA, Payen JF, Pun BT, Puntillo KA, Riker RR, Robinson BR, Shehabi Y, Szumita PM, Winkelman C, Centofanti JE, Price C, Nikayin S, Misak CJ, Flood PD, Kiedrowski K, Alhazzani W (2018). Clinical practice guidelines for the prevention and management of pain, agitation/sedation, delirium, immobility, and sleep disruption in adult patients in the ICU. Crit Care Med.

[15] Lang JK, Paykel MS, Haines KJ, Hodgson CL (2020). Clinical practice guidelines for early mobilization in the ICU: a systematic review. Crit Care Med.

[16] Morandi A, Brummel NE, Ely EW (2011). Sedation, delirium and mechanical ventilation: the 'ABCDE' approach. Curr Opin Crit Care.

[17] Pandharipande P, Banerjee A, McGrane S, Ely EW (2010). Liberation and animation for ventilated ICU patients: the ABCDE bundle for the back-end of critical care. Crit Care.

[18] Barnes-Daly MA, Phillips G, Ely EW (2017). Improving hospital survival and reducing brain dysfunction at seven California community hospitals: implementing PAD guidelines via the ABCDEF bundle in 6,064 patients. Crit Care Med.

[19] Balas MC, Vasilevskis EE, Olsen KM, Schmid KK, Shostrom V, Cohen MZ, Peitz G, Gannon DE, Sisson J, Sullivan J, Stothert JC, Lazure J, Nuss SL, Jawa RS, Freihaut F, Ely EW, Burke WJ (2014). Effectiveness and safety of the awakening and breathing coordination, delirium monitoring/management, and early exercise/mobility bundle. Crit Care Med.

[20] Pun BT, Balas MC, Barnes-Daly MA, Thompson JL, Aldrich JM, Barr J, Byrum D, Carson SS, Devlin JW, Engel HJ, Esbrook CL, Hargett KD, Harmon L, Hielsberg C, Jackson JC, Kelly TL, Kumar V, Millner L, Morse A, Perme CS, Posa PJ, Puntillo KA, Schweickert WD, Stollings JL, Tan A, D'Agostino McGowan L, Ely EW (2019). Caring for critically ill patients with the ABCDEF bundle: results of the ICU liberation collaborative in over 15,000 adults. Crit Care Med.

[21] Costa DK, Barg FK, Asch DA, Kahn JM (2014). Facilitators of an interprofessional approach to care in medical and mixed medical/surgical ICUs: a multicenter qualitative study. Res Nurs Health.

[22] Needham DM, Korupolu R (2010). Rehabilitation quality improvement in an intensive care unit setting: implementation of a quality improvement model. Top Stroke Rehabil.

[23] Schallom M, Tymkew H, Vyers K, Prentice D, Sona C, Norris T, Arroyo C (2020). Implementation of an interdisciplinary AACN early mobility protocol. Crit Care Nurse.

[24] Schaller SJ, Anstey M, Blobner M, Edrich T, Grabitz SD, Gradwohl-Matis I, Heim M, Houle T, Kurth T, Latronico N, Lee J, Meyer MJ, Peponis T, Talmor D, Velmahos GC, Waak K, Walz JM, Zafonte R, Eikermann M, International Early SOMS-guided Mobilization Research Initiative (2016). Early, goal-directed mobilisation in the surgical intensive care unit: a randomised controlled trial. Lancet.

[25] Phelan S, Lin F, Mitchell M, Chaboyer W (2018). Implementing early mobilisation in the intensive care unit: an integrative review. Int J Nurs Stud.

[26] Krupp AE, Ehlenbach WJ, King B (2019). Factors nurses in the intensive care unit consider when making decisions about patient mobility. Am J Crit Care.

[27] Drews FA (2013). Human factors in critical care medical environments. Rev Hum Factors Ergon.

[28] Sanchez-Pinto LN, Luo Y, Churpek MM (2018). Big data and data science in critical care. Chest.

[29] Sutton RT, Pincock D, Baumgart DC, Sadowski DC, Fedorak RN, Kroeker KI (2020). An overview of clinical decision support systems: benefits, risks, and strategies for success. NPJ Digit Med.

[30] Khairat SS, Dukkipati A, Lauria HA, Bice T, Travers D, Carson SS (2018). The impact of visualization dashboards on quality of care and clinician satisfaction: integrative literature review. JMIR Hum Factors.

[31] Kortebein P, Symons TB, Ferrando A, Paddon-Jones D, Ronsen O, Protas E, Conger S, Lombeida J, Wolfe R, Evans WJ (2008). Functional impact of 10 days of bed rest in healthy older adults. J Gerontol A Biol Sci Med Sci.

[32] English KL, Paddon-Jones D (2010). Protecting muscle mass and function in older adults during bed rest. Curr Opin Clin Nutr Metab Care.

[33] Dunn Lopez K, Gephart SM, Raszewski R, Sousa V, Shehorn LE, Abraham J (2017). Integrative review of clinical decision support for registered nurses in acute care settings. J Am Med Inform Assoc.

[34] Krupp A, Potter K, Wendt L, Dunn Lopez K, Dunn H (2025). Using electronic health records to classify risk for adverse safety events with ICU patient mobility: a cross-sectional study. Intensive Crit Care Nurs.

[35] Wasylewicz AT, Scheepers-Hoeks AM, Kubben P, Dumontier M, Dekker A (2018). Clinical decision support systems. Fundamentals of Clinical Data Science.

[36] Hodgson CL, Stiller K, Needham DM, Tipping CJ, Harrold M, Baldwin CE, Bradley S, Berney S, Caruana LR, Elliott D, Green M, Haines K, Higgins AM, Kaukonen KM, Leditschke IA, Nickels MR, Paratz J, Patman S, Skinner EH, Young PJ, Zanni JM, Denehy L, Webb SA (2014). Expert consensus and recommendations on safety criteria for active mobilization of mechanically ventilated critically ill adults. Crit Care.

[37] Dunn H, da Costa Ferreira Oberfrank N, Krupp A (2024). Preimplementation of critical care early mobility clinical decision support: a content validation study. Comput Inform Nurs.

[38] Holden RJ, Karsh BT (2010). The technology acceptance model: its past and its future in health care. J Biomed Inform.

[39] Tao D, Wang T, Wang T, Zhang T, Zhang X, Qu X (2020). A systematic review and meta-analysis of user acceptance of consumer-oriented health information technologies. Comput Hum Behav.

[40] Venkatesh V, Speier C, Morris MG (2007). User acceptance enablers in individual decision making about technology: toward an integrated model. Decis Sci.

[41] Ayres P, Paas F (2012). Cognitive load theory: new directions and challenges. Appl Cogn Psychol.

[42] Paas F, Renkl A, Sweller J (2004). Cognitive load theory: instructional implications of the interaction between information structures and cognitive architecture. Instr Sci.

[43] Hart SG, Staveland LE, Hancock PA, Meshkati N (1988). Development of NASA-TLX (task load index): results of empirical and theoretical research. Advances in Psychology.

[44] Damschroder LJ, Reardon CM, Widerquist MA, Lowery J (2022). The updated consolidated framework for implementation research based on user feedback. Implement Sci.

[45] Damschroder LJ, Aron DC, Keith RE, Kirsh SR, Alexander JA, Lowery JC (2009). Fostering implementation of health services research findings into practice: a consolidated framework for advancing implementation science. Implement Sci.

[46] Nielsen J (1992). The usability engineering life cycle. Computer.

[47] Jha A, Suarez ML, Ferrans CE, Molokie R, Kim YO, Wilkie DJ (2010). Cognitive testing of PAINReportIt in adult African Americans with sickle cell disease. Comput Inform Nurs.

[48] Makri S, Blandford A, Cox AL (2011). This is what I’m doing and why: methodological reflections on a naturalistic think-aloud study of interactive information behaviour. Inf Process Manag.

[49] Holden RJ, Brown RL, Scanlon MC, Karsh BT (2012). Modeling nurses' acceptance of bar coded medication administration technology at a pediatric hospital. J Am Med Inform Assoc.

[50] Lopez KD, Febretti A, Stifter J, Johnson A, Wilkie DJ, Keenan G (2017). Toward a more robust and efficient usability testing method of clinical decision support for nurses derived from nursing electronic health record data. Int J Nurs Knowl.

[51] Krupp A, Di Martino M, Chung W, Chaiyachati K, Agarwal AK, Huffenberger AM, Laudanski K (2021). Communication and role clarity inform TeleICU use: a qualitative analysis of opportunities and barriers in an established program using AACN framework. BMC Health Serv Res.

[52] Benner P, Tanner C, Chesla C (1992). From beginner to expert: gaining a differentiated clinical world in critical care nursing. ANS Adv Nurs Sci.

[53] Benner P (2004). Using the Dreyfus model of skill acquisition to describe and interpret skill acquisition and clinical judgment in nursing practice and education. Bull Sci Technol Soc.

[54] Dillman DA, Smyth JD, Christian LM (2014). Internet, Phone, Mail, and Mixed‐Mode Surveys: The Tailored Design Method. 4th edition.

[55] Davis FD (1989). Perceived usefulness, perceived ease of use, and user acceptance of information technology. MIS Q.

[56] Weiner BJ, Lewis CC, Stanick C, Powell BJ, Dorsey CN, Clary AS, Boynton MH, Halko H (2017). Psychometric assessment of three newly developed implementation outcome measures. Implement Sci.

[57] Brooke J, Jordan PW, Thomas B, McClelland IL, Weerdmeester B (1996). SUS: a 'quick and dirty' usability scale. Usability Evaluation in Industry.

[58] Brooke J (2013). SUS: a retrospective. J Usability Stud.

[59] Tubbs-Cooley HL, Mara CA, Carle AC, Gurses AP (2018). The NASA Task Load Index as a measure of overall workload among neonatal, paediatric and adult intensive care nurses. Intensive Crit Care Nurs.

[60] Glasgow RE, Harden SM, Gaglio B, Rabin B, Smith ML, Porter GC, Ory MG, Estabrooks PA (2019). RE-AIM planning and evaluation framework: adapting to new science and practice with a 20-year review. Front Public Health.

[61] Hodgson CL, Bailey M, Bellomo R, Brickell K, Broadley T, Buhr H, Gabbe BJ, Gould DW, Harrold M, Higgins AM, Hurford S, Iwashyna TJ, Serpa Neto A, Nichol AD, Presneill JJ, Schaller SJ, Sivasuthan J, Tipping CJ, Webb S, Young PJ, TEAM Study Investigators and the ANZICS Clinical Trials Group (2022). Early active mobilization during mechanical ventilation in the ICU. N Engl J Med.

[62] Lopez KD, Fahey L (2018). Advocating for greater usability in clinical technologies: the role of the practicing nurse. Crit Care Nurs Clin North Am.

[63] Mao JY, Vredenburg K, Smith PW, Carey T (2005). The state of user-centered design practice. Commun ACM.

[64] Campese C, Amaral DC, Mascarenhas J (2020). Restating the meaning of UCD and HCD for a new world of design theories. Interact Comput.

[65] Trinkley KE, Kroehl ME, Kahn MG, Allen LA, Bennett TD, Hale G, Haugen H, Heckman S, Kao DP, Kim J, Matlock DM, Malone DC, Page Nd RL, Stine J, Suresh K, Wells L, Lin CT (2021). Applying clinical decision support design best practices with the practical robust implementation and sustainability model versus reliance on commercially available clinical decision support tools: randomized controlled trial. JMIR Med Inform.

[66] Linke CA, Chapman LB, Berger LJ, Kelly TL, Korpela CA, Petty MG (2020). Early mobilization in the ICU: a collaborative, integrated approach. Crit Care Explor.

[67] Yao Y, Dunn Lopez K, Bjarnadottir RI, Macieira TG, Dos Santos FC, Madandola OO, Cho H, Priola KJ, Wolf J, Wilkie DJ, Keenan G (2023). Examining care planning efficiency and clinical decision support adoption in a system tailoring to nurses' graph literacy: national, web-based randomized controlled trial. J Med Internet Res.

