# Supplementary material for: Aggregating Patient Safety and Status Information in the Electronic Health Record to Support Time-Sensitive Mobility Interventions in the Intensive Care Unit: Protocol for the Design and Testing of a Clinical Decision Support Tool
Source: JMIR Res Protoc. 2025 Oct 16;14:e75752. doi: 10.2196/75752 (PMC12576298; doi:10.2196/75752)
Supplement: Multimedia Appendix 1 [file resprot_v14i1e75752_app1.docx]

**ASSIST-ICU User Centered Design Interview Guide (Phase 1)**

**Orientation to the study**

**Welcome and introductions**

*Interviewer:* Hi  __________, thank you so much for coming today.  My name is ___________ and I am going to be working with you today during this session to review a new EHR tool focused on patient mobility in the ICU. We will explain more about the tool as we continue our discussion today.

**Agenda**

*Interviewer:* Here is the agenda for today. We will start by introducing our team members, talk about any of the survey's and permission forms that may still need to be completed, provide a short overview of what you will be doing in this session, discuss the interview process, let you explore our tool and give feedback, and then have one final survey to complete.

We will start with the introduction of our team members *(Pause for introductions)*

*Interviewer:* We are going to start with a couple of short online surveys and forms.

**Surveys & Permissions**

*(If pre-surveys already completed)*

*Interviewer:* Thank you for completing the demographics and compensation info ahead of time, we appreciate that.

Please go ahead and click on the link to open that survey.

*(If pre-surveys not completed)*

I’m going to give you a link in the chat that will take you to a demographics survey. Please go ahead and open that survey. You will see some demographics and work history questions to answer.

The last question is acknowledging that we will be making audio and screen recordings of your interview to best track and understand how the electronic health record tool is utilized.

Next you will see a compensation info form. The information requested here is necessary for compensation and allows the University of Iowa to meet government reporting obligations. If you do not want to provide this information you can decide to not receive payment for funding.

**Early Mobility**

*Interviewer:* Next, I want to introduce mobility in ICU settings.

As you may know, there is some evidence that ICU patients who receive mobility interventions have better outcomes than patients who do not. However, it can be difficult to determine which patients are safe to mobilize. The tool we are going to have you review was built with this foundational knowledge.

**Overview**

*Interviewer:* Next, I will give you an overview of what we will be doing during this interview.  The first part of our study involves looking at the tool we have developed and exploring this tool. This process is called a "Think aloud" interview which may be a little bit different than other interviews you may have experienced. We will then provide a unit context and report on 2 fictitious patients and if you would like to take notes as you would in an actual report that is fine with us as you may want to have those details when you are looking at the tool.

Once we have prepped you with the scenario, we will put a link in the chat and ask that you share your screen. The recording will begin once you have shared your screen and the think aloud interview will then begin. Do you have any questions?

**Part 1: Think Aloud Interviews**

*Interviewer:* Let’s start by talking about think alouds. One way that we can learn about how you use our tool is by having you think out loud while you are exploring the tool. For the think aloud interview, once we have given you the link and you have opened it, we would like you to explore the interactive screen (or interface). This view mimics an electronic health record screen that ICU clinicians would have access to as part of their routine workflow. While you are exploring, we would like you to “think aloud” or talk about what you are looking at on the screen, what you are trying to find on the screen and/or what you are thinking about.

**Think Aloud Example**

For example: “This view makes me think the patient is doing well but I wonder what else I can find out about the patient. Maybe if I click in the upper left area…” This “think aloud” or talking out loud interview will be very helpful to us.  It is also helpful if you are specific when you mention the parts of the screen you are talking about, for example say aloud, “the circle in the middle” I will try not to interrupt you and most of the time we will be listening to you talk. I realize it is unnatural for most people to talk about what you are doing as they are doing it, if we notice you are not talking enough, one of our team members may remind you to talk more. Your ideas will help our design team make it better, I want to emphasize that you should not be afraid to be critical of the tool.

**Scenario Overview**

*Interviewer:* To help set the stage for how and why you might access our new tool, I will be giving you a fictitious setting and data about two patients you are caring for.  While you are exploring the tool, I want you to imagine you are a nurse on this unit, working a 12-hour shift 0700 to 1900 on July 5th.  You are caring for two patients whose data are provided to you on the upcoming slides.

**Unit Context**

*Interviewer:* The unit context is a 12 bed Mixed ICU. The most common patient admissions include sepsis, respiratory failure, heart failure, and GI bleed. There are 6 nurses per shift and a charge nurse. The charge nurse may be assigned one patient. One nursing assistant is assigned per shift for the whole unit. Currently there are no traveler nurses staffing in the unit. There is one ICU intensivist-led team covers all patients. The team includes a nurse practitioner. One PT covers all ICU consults Monday-Friday. The PT may also see patients in the burn unit. One RT is dedicated to the ICU each shift. Today, the ICU is full. There are 3 planned discharges and one planned ICU admission.

**Unit Culture**

*Interviewer:* On our fictitious unit, they have implemented a nurse-directed early mobility protocol, which provides screening criteria for nurses to help determine whether a patient is clinically ready to begin out-of-bed mobility. The unit’s culture related to patient mobility is that mobility is a multi-professional intervention, early mobility is provided to all patients who are deemed safe following the unit’s screening protocol, including patients who are mechanically ventilated.

**Mobility Protocol**

*Interviewer:* This is the unit specific criteria for early mobilization. (*Wait for participant to read the protocol.)*

**Patient Assignment #1**

*Interviewer:* The first patient you will be taking care of is Jill Roman. Here is some of the information provided in report:

*(once done looking at information)*

*Interviewer:* Your assessment is consistent with [RN: handoff report/ PT: chart review]. Jill is following commands. When testing muscle strength in lower extremities, patient actively moves against gravity and resistance.

**Patient Assignment #2**

*Interviewer:* Patient #2 is Carlos Baker. Here is some of the information provided in report for your second patient Carlos Baker (Give time to look at slide)

*Interviewer:* Your first assessment is consistent with [RN: handoff report/ PT: chart review]. Carlos is following commands and actively moving in bed. When testing his muscle strength in lower extremities you find that the patient actively moves against gravity and resistance. This is not the first mobilization attempt for either patient.

With these two fictitious patients in mind, right now, we would like you to explore these screens in the way that you want to assess readiness to mobilize today. Do you have questions or need to take a short break?

**Prototype Link**

*Interviewer:* We will give you a link in the chat to an interactive screen (or interface) that mimics an electronic health record screen that ICU clinicians would have access to as part of their routine workflow. I will give you that link now. Once you have it open, we need you to share your screen, and we will begin recording.

Now we would like you to explore the interface. While you are exploring, we would like you to “think aloud” or talk about what you are looking at on the screen, what you are trying to find on the screen and/or what you are thinking about. It’s very important that you know that you will not offend the research team if you find weaknesses in the tool.

Thank you for explaining your thinking. Now we’d like to ask some additional questions.

**Prototype V1 Cognitive Interview Questions**

**Sample questions to complete for each patient display:**

1. Based on what you have seen, what would be your decision about mobilizing this patient?
   1. Ok, tell me about what factors help you make that decision?
2. Let’s talk about the graphs for a bit. *Example questions, tailored to specific features:*
   1. What’s the meaning or your interpretation of the graph in the upper left corner?
   2. What do you think the green line means?
   3. What about the yellow line?
   4. What does the red line changing to blue convey to you?
   5. What does the trend line mean to you?
3. Is there any additional information you need?
4. Is there any information that we don’t need or could get rid of?

**Sample questions to complete for overall display:**

1. When you first opened the link, the first view is of the overall unit information, is there any information missing from this view?
2. From a design standpoint, some people have not perceived that the mobility score button was interactive.  Do you have any suggestions to make it clear that the mobility button is interactive?

**Sample questions for overall design ideas:**

1. Thinking about the two different patient dashboards, do you prefer one over the other?  If so, why?
2. Is there anything that seemed confusing when using the tool?
3. Is there anything that you had hoped you would see but didn’t?
4. Is there anything else you would like to share about our tool?
