# Supplementary material for: Aggregating Patient Safety and Status Information in the Electronic Health Record to Support Time-Sensitive Mobility Interventions in the Intensive Care Unit: Protocol for the Design and Testing of a Clinical Decision Support Tool
Source: JMIR Res Protoc. 2025 Oct 16;14:e75752. doi: 10.2196/75752 (PMC12576298; doi:10.2196/75752)
Supplement: Multimedia Appendix 2 [file resprot_v14i1e75752_app2.docx]

**ASSIST-ICU Implementation Planning Interview Guide (Phase 1)**

**Introduction:** My name is ________, and I am a [role] on a research team aiming to develop and test a new clinical decision support tool in the electronic health record for ICU RNs and PTs to support decision-making about early mobility. Our team is interested in your experiences as a [role], and we would like to learn more about what’s worked well with implementing practice changes that involve the electronic health record and potential challenges prior implementing our tool.

Your participation will involve one informal interview that will last between 30 and 60 minutes. This research has no known risks. This research will benefit the academic community because it helps us understand barriers and facilitators to implementing a new clinical decision support tool in the electronic health record.

Please know that I will do everything I can to protect your privacy. Your identity or personal information will not be disclosed in any publication that may result from the study. Notes that are taken during the interview will be stored in a secure location. We would like to record this meeting so that we don’t lose any of the information discussed. Would you be ok if we record this conversation?

Do you have any questions before we begin?

**INTERVIEW QUESTIONS**

1. What is your current role and how long have you been in this position?

*The following questions are based on the Consolidated Framework for Implementation Research (CFIR) and questions will be selected and tailored based upon interviewee role.*

| **Domain** | **Construct** | **Question** |
| --- | --- | --- |
| Inner Setting | Work Infrastructure | 1. How do RNs and PTs currently organize mobility interventions for patients in your unit? Are there differences during the week vs. weekend? |
|  | Communication | 1. How do RNs and PTs currently communicate about mobility plans for a patient? 2. How do [informaticists/quality leaders] and ICU clinicians communicate? |
|  | Culture | 1. What are current [RN/PT] attitudes about practice change in your unit? 2. What are current [RN/PT] attitudes about clinical decision support in your unit? 3. What are current [RN/PT] attitudes and practices around mobility in your unit? |
|  | Information Technology Infrastructure | 1. What types of CDSS do [RNs/PTs] currently use in practice? 2. How do [RNs/PTs] learn about new ITS tools, such as CDSS? 3. What encourages [RNs/PTs] to try and use new ITS tools, such as CDSS? |
|  | Compatibility | 1. What is the current workflow for an [RN/PT] in deciding to engage a patient in out-of-bed mobility? When do they engage with the EHR? 2. What is the current process for implementing a new CDSS for a unit or service line? |
|  | Available Resources | 1. What resources are needed to implement CDSS? 2. What is the current process for ongoing CDSS review after implementation (e.g., use, troubleshooting)? |
| Implementation Process |  | 1. How are new tools in the EHR typically implemented? 2. What is the typical timeframe? 3. What training has been helpful? 4. Are their challenges you’ve experienced with implementing CDSS? |

**WRAP UP**

1. Do you have any additional suggestions for us as we move forward with this study?
2. Are there other individuals or roles you think it would be important for us to talk with?

Thank you for your time.
